# Measuring the Pores’ Structure in P3HT Organic Polymeric Semiconductor Films Using Interface Electrolyte/Organic Semiconductor Redox Injection Reactions and Bulk Space-Charge

**DOI:** 10.3390/polym14173456

**Published:** 2022-08-24

**Authors:** Franz Schauer

**Affiliations:** Faculty of Applied Informatics, Tomas Bata University in Zlin, Nad Stranemi 4511, 760 05 Zlin, Czech Republic; fschauer@fai.utb.cz

**Keywords:** organic polymeric semiconductors, pores structure, electronic structure, redox reactions

## Abstract

The article is another in a series of follow-up articles on the new spectroscopic method Energy Resolved–Electrochemical Impedance Spectroscopy (ER-EIS) and presents a continuation of the effort to explain the method for electronic structure elucidation and its possibilities in the study of organic polymeric semiconductors. In addition to the detailed information on the electronic structure of the investigated organic semiconductor, the paper deals with three of the hitherto not solved aspects of the method, (1) the pores structure, which has been embedded in the evaluation framework of the ER-EIS method and shown, how the basic quantities of the pores structure, the volume density of the pores’ density coefficient *β* = (0.038 ± 0.002) nm^−1^ and the Brunauer-Emmet-Teller surface areas SABET SA == 34.5 m^2^g^−1^ may be found by the method, here for the archetypal poly(3-hexylthiophene-2,5-diyl) (P3HT) films. It is next shown, why the pore’s existence needs not to endanger the spectroscopic results of the ER-EIS method, and a proper way of the ER-EIS data evaluation is presented to avoid it. It is highlighted (2), how may the measurements of the pore structure contribute to the determination of the, for the method ER-EIS important, real rate constant of the overall Marcus’ D-A charge-transfer process for the poreless material and found its value k*_ct_*_D-A_ = (2.2 ± 0.6) × 10^−25^ cm^4^ s^−1^ for P3HT films examined. It is also independently attempted (3) to evaluate the range of k*_ct_*_D-A_, based on the knowledge of the individual reaction rates in a chain of reactions, forming the whole D-A process, where the slowest one (organic semiconductor hopping transport) determines the tentative total result k*_ct_*_D-A_ ≅ 10^−25^ cm^4^ s^−1^. The effect of injection of high current densities by redox interface reactions in the bulk of OS with built-in pores structure may be very interesting for the design of new devices of organic electronics.

## 1. Introduction

Organic semiconductors (OS) are electronically conducting polymers, which differ from their inorganic counterparts by extrinsic conductivity, resulting in the injection of charges at electrodes, by intentional or unintentional doping, and by the dissociation of photogenerated electron-hole pairs that are bound by their mutual Coulomb attraction. The underlying physics of organic semiconductors is thus very different from that of their inorganic analogs, but the laws of physics are very same, yet some parameters take on different values [1]. Moreover, the fact that in disordered organic semiconductors the charge-transporting moieties are coupled by weak van der Waals force has two important consequences, first, when abandoning the crystallographic order in a molecular crystal, no bond breaking occurs, that is, there are no dangling bonds, and surface states and consequently no pinning of the Fermi energy can occur, second, the valence and conduction bands are completely decomposed into a manifold of localized states and charge carriers hop randomly between the structural elements [1].

**Figure 1:**
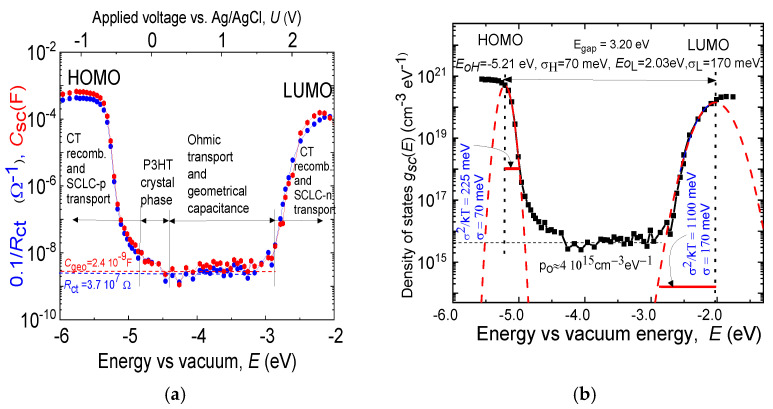
ER-EIS spectra of P3HT film, *L* = 300 nm (**a**) The rough unprocessed ER-EIS data plotted as 0.1/*R_ct_* and *C_sc_* vs. applied voltage *U* and energy towards vacuum *E*; (**b**) The bulk DOS function *g_sc_*(*E*) (black) (calculated from (**a**) and Equation (3)) with the denoted Gaussian distributions (red).

The electronic structure of disordered organic semiconductors is used, in connection with the statistics of neutral and charged states, to explain on the microscopic level the charge transport, trapping, and recombination using the Gaussian disorder model, also known as the Bässler model [2]. It is based upon the notion that charge carriers hop within a manifold of sites that feature a Gaussian energy distribution and Gaussian distribution of inter-site spacing (see dashed red lines in Figure 1b),$\;g\left(E\right)=\frac{N}{\;\sqrt{2\pi }\;\sigma }\;e^{\left[\frac{-{\left(E-E_{o}\right)}^{2}}{2\sigma^{2}}\right]}$, where *N* is the total concentration of states (structural units), *E* is the energy of an individual molecule, *E_o_* is the peak of the distribution and the standard deviation *σ* (as a disorder parameter), characterizes the width of the Gaussian distribution.

The equivalent to the valence and conduction bands in inorganic solids emerges HOMO and LUMO as the highest occupied and the lowest unoccupied molecular orbitals, respectively. They are separated by the transport gap, also called the single-particle gap. The electronic structure of intrinsic and extrinsic defects in organic semiconductors is a major topic in organic electronics. In any microelectronic device, fundamental physical parameters including the electron structure of defects must be well understood for successful electronic optimization [3].

To understand the behavior of the OS with the Gaussian DOS distribution, both modeling and measuring using spectroscopical methods are required. It is proper to mention, that any close solutions for full occupation statistics of the Gaussian distribution do not exist and we have to revert to the numerical modeling to know its behavior. The second source of possible knowledge about the Gauss distribution is suitable spectroscopy. Unfortunately, the researcher in OS is limited to the methods albeit suffering from low dynamics and/or a limited energy span.

Recently, there have been demands for new methods for a wide energy range [4] and accuracy [5] These needs meet a new spectroscopic method Energy-Resolved Electrochemical Impedance Spectroscopy (ER-EIS) for the elucidation of the electronic structure of OS [6]. In solid-state physics, there has never been a situation, where the characterization of any important microphysical quantity was entrusted to the electrochemistry and the Helmholtz double-layer (HDL) [7]. The reason could be the innate shyness of physicists to electrolytes and measurements in them. It is possible to demonstrate this on a spectroscopic method ER-EIS and such a quantity as the electronic structure of disordered organic semiconductors, so perspective in terms of applications. The method ER-EIS represents the use of both the electrochemical phenomenon given by the outstanding properties of the HDL, forming and forcing the current through the system electrolyte/OS by the mechanism of redox reactions at the interface, leading to the electronic transport of space-charge limited currents in the bulk of the OS, exploiting the barrierless contacts to the OS bulk via redox reactions. An extreme advantage that added value is that although the two phenomena (interface and bulk) are interrelated, they provide independent information about the same quantity both at the surface and the volume of the OS in a broad energy span with a large dynamic range. Organic disordered semiconductors possess several exceptional qualities for electronic structure elucidation: the Fermi energy position is governed by the well-defined energy of dominating states in the continuum of localized states distributed in energy both at the surface and in the bulk of the OS. Next, the time constants for charge carriers’ transport in the bulk of the OS are many orders lower than the diffusion transport time constants, so they prevail in a time–domain of the whole charge-transfer D-A process, in contradiction to their inorganic counterparts, where it is reversed. The method has been already used with success on the OS and their blends characterization [8,9,10,11], their degradation [12,13] and also on the films of nanoparticles [14].

Still, several points, connected to the ER-EIS method for real-world use are worth deeper insight and explanation, and some of them are the topic of the present paper. Before all it is the point of the pore structure existence, the problem of the role of the omnipotent compensating ions in conductive OS with HDL and its influence on the ER-EIS data and what we may learn from it.

The paper is organized in the following way. First, the paper deals in more detail with the possible influence of the pore structure existence on the experimental results of the method ER-EIS. Second, based on the model of electron transfer in the system electrolyte/OS, it is attempted to search for the overall rate constant of the whole-round donor-acceptor transport including redox recombination at the interface electrolyte/OS. Third, some argument is provided for the often-spelled objection towards electrochemical methods with solids, the ill role of compensating ions penetrating the solid material and influencing by their charge: the Fermi energy and, by their size: the morphology of the polymer. Fourth, it is attempted to present the overall rate constant of D-A transfer, based on Marcus’ original based on the model of the series of steps with the predominant slow step by the hopping transport in OS.

## 2. Methods and Materials

### 2.1. ER-EIS for the Electronic Structure Spectroscopy of Real Organic Semiconductors

Let us highlight the short cross-section of the spectroscopic method ER-EIS (details to be found in [6] with the supposition of poreless OS) with typical examples of its use and a short description of samples preparation. The electrochemical method ER-EIS is based on the measurements of the small-signal impedance as a response to the perturbance by the AC signal of the frequency ω, *Ẑ*(*E*_F_) = *R_ct_* + 1/j*ωC_sc_*, whose real and imaginary parts are the differential charge-transfer (CT) resistance *R_ct_*(*E*_F_), characterizing the OS surface, and the differential space-charge capacitance *C_sc_*(*E*_F_)_,_ characterizing the OS bulk. The position of the Fermi energy *E*_F_= −e*V*_Pt_ is adjusted by the external voltage (*U* = *V*_Pt_).

Figure 1a is information-rich rough ER-EIS data of P3HT film in the format 0.1/*R_ct_* and *C_sc_*, spanning in energy vs. vacuum *E*€(−6; −2)eV and covering the dynamics of both quantities by more than 5 orders of magnitude. The signals stem both from the HOMO band with reduction and the LUMO with oxidation process of charged localized states of OS (or in other words occupying with electron or removing electron to (from) the surface states of the OS, respectively). The doping (injection) is going from the zero voltage *U* = 0 V, applied to the Pt electrode, to negative voltages (*U* < 0 V), when scanning the HOMO band, whereas to the positive (*U* > 0 V) voltages, while scanning the LUMO band.

It is remarkable, that both experimental data in Figure 1a, though stemming from the diverse phenomena, at either the electrolyte/OS interface (*R_ct_*) or in the bulk of the OS (*C_sc_*), give approximately $\frac{0.1}{R_{ct}\left(E_{F}\right)}=C_{sc}\left(E_{F}\right)$. This highlights the approximate fulfillment of two basic conditions for the ER-EIs method, the first is, the necessary impedance phase shift of 45° and the measuring angular frequency *ω* for the whole span of external voltages fulfilling

(1) $$R_{ct}\left(E_{F}\right)C_{sc}\left(E_{F}\right)=\omega^{-1}$$

and the second, the dependences of both quantities *R_ct_* and *C_sc_* on equal concentrations of charge carriers (holes—for HOMO or electrons for LUMO) at the surface of OS *R_ct_* ≈ 1/*p*_surf_ and in its bulk and *C_sc_* ≈ *p_sc_* with their constant product (more details to be found in [6]).

The measured differential space-charge capacitance *C_sc_*

(2) $$C_{sc\left(E_{F}\right)}=e\;g_{sc}\left(E_{F}\right)LS_{o}\;$$

is the only source of ER-EIS data for electronic structure-function *g_sc_*(*E*_F_) evaluation without any additional constants

(3) $$g_{sc}\left(E_{F}\right)=\frac{C_{sc\left(E_{F}\right)}}{e\;S_{o}L}$$

where

$$C_{sc}\left(E_{F}\right)=LS_{o}\frac{\;\partial q_{sc}}{\partial U_{\mathrm{SCLC}}}$$

*S_o_* is the surface area of OS film and *L* its thickness, and *C_sc_* scales with the volume of the sample. Figure 1b depicts the result of the evaluation for electronic structure, using the imaginary component of the measured impedance, here denoted as capacitance *C_sc_* and using Equation (3). 

The measured CT differential resistance as a measured real part of the impedance, defined as ${\left[R_{ct}\left(E_{F}\right)\right]}^{-1}=S\frac{\;\partial j_{rec}}{\partial U}$ is [15]

(4) $$\;{\left[R_{ct}\left(E_{F},L\right)\;\right]}^{-1}=e\;S_{o}\;\left[A\right]\;g_{ct\;}\left(E_{F}\right)\;k_{ct\;}$$

here [*A*] is the active concentration of the ionic charges dissolved in the solvent, *g_ct_*(*E*) (cm^−3^ eV^−1^) is the DOS of the electronic states at the surface of OS, and *k_ct_* (cm^4^ s^−1^) is the rate constant for the whole D-A process. Equation (4). cannot be used directly for the evaluation of the DOS function *g_ct_*(*E*) due to the lack of data on the rate constant *k_ct_*. 

The transfer rate function and constant constitute a rather complicated problem of quantum physics, solved completely for the diffusion-governed process for the interface electrolyte/crystalline semiconductor [15]

(5) $$k_{ct\;}\left(E\right)=k_{ct\;max}\;exp\left[\frac{-{\left(E_{redox}-E+\lambda \right)}^{2}}{4\lambda kT}\right]{,}^{\;}$$

where *λ* is the reorganization energy. It was shown by Wang et al. [16] suggest for *E* ≪ *λ* the Equation (5) reduces to a constant, *k_ct_* = *k_ct max_* independent of energy. The theoretical conclusions of Marcus [17,18] were confirmed on crystalline semiconductors in the paper by Lewis [19], aligning both semi-classical and quantum calculations with the experiment. The experiment gave *k_et max_* in the interval of 10^−16^–10^−18^ cm^4^ s^−1^ for both measured electrolyte/crystalline semiconductor interfaces with Si [20] and InP [21]. Unfortunately, till now, critical structure-property relationships in electrochemical systems with a disordered OS have been derived only for a few materials only, and the rate constant for the reaction limited process for the system electrolyte/OS has not been, to our knowledge, derived or measured yet.

### 2.2. Is the Existence of Pores Influencing the ER-EIS Results?

Pores’ existence and pores’ structure are inevitable in organic conductive polymers [22] and thus widely studied with respect to the increasing the apparent surface of the solid material, relevant to polymer batteries, energy storage, and organic electronics in general [23]. The pore radius in microporous polymers is generally about 1–2 nm [24], studied by many experimental methods including nitrogen sorption porosimetry [25] with the output of apparent Brunauer-Emmet-Teller surface areas SA_BET_ and ellipsometry [26]. Microporous conductive polymers with SA_BET_ = 1046 m^2^/g were produced for polysilanesTetrakis(4-bromophenyl) silane (TBPS) [27] and Poly(phenylenevinylene) (PPV) *S*A_BET_ = 761 m^2^g^−1^ [28] to give examples, pertinent to this study.

Let us imagine the ER-EIS electrode with the microporous polymer of the geometrical surface area *S*_o_ and the thickness *L* with pores, filled with electrolyte and applied voltage *V*_Pt_ < 0. Modeling real electrodes with pores is a demanding task, a simpler model is usually used, in which it is assumed that pores have a cylindrical shape with a length *L* and a radius *r* and, as is highlighted in [29] the states at the surface of the optimized polymer are more or less identical to those in the bulk, and thus it is probable the walls of the pores behave similarly in redox processes like the free surface of the sample.

For the method ER-EIS is decisive the impedance *Ẑ* of the individual pore of a porous electrode was calculated first by De Levie [30]. The real part of the impedance, *R_ct_*, then for thin pores with *r* ≈ 1–2 nm, the absence of internal diffusion in electrolyte towards the wall of the pore and the penetration length *λ* of the AC signal into the pore, fulfilling *λ* >> *L*. We have for the effective area of a single pore *S*_p_ = 2π*rL* >> πr^2^ and if we suppose the density of pores per unit area n, *S_o_*n is the total number of pores in the sample and *S*_o_*β* is the increase of the effective area of the sample per unit length, and finally *S*_o_*βL = SS*_o_ is the effective area increase of the sample due to the pores’ existence, where we introduced the coefficient *β* = n2π*r* ([*β*] = cm^−1^).

The measured differential space-charge capacitance *C_sc_* is not influenced by the existence of the pores and Equations (2) and (3) are used for evaluation of DOS without any change, as the area *S*_o_ for SCLC current transport is not changed, due to small dimensions of the pores.

In opposite, also measured differential resistance *R_ct_*(*E*_F_) is influenced by the existence of pores via the increase of the effective reaction area of the sample *S = S*_o_ *+ S*_o_*βL,* changing Equation (4). to

(6) $$\;{\left[R_{ct}\left(E_{F}\right)\;\right]}^{-1}=eS_{o}\;\left(1+\beta L\right)\;\left[A\right]\;g_{ct\;}\left(E_{F}\right)\;k_{ct\;}.$$

It is obvious from Equation (6) that the measured *R_ct_*(*E*_F_) data alone cannot be used for the determination of the DOS *g_ct_*(*E*_F_) function, as it contains two unknown quantities, the pores’ coefficient *β*, and the rate constant *k_ct_*_._ To get from the ER- EIS data of all three unknown quantities, the electron structure DOS function *g_ct_*(*E*), the pores’ density coefficient *β*, and the transfer rate preexponential constant *k_ct_*, we have to resort to the evaluation of the thickness measurements referred in Chapter Results. 

### 2.3. Useful ER-EIS Outputs—Effective Rate Constant

Equation (6) may be reformulated

(7) $$\;{\left[R_{ct}\left(E_{F},L\right)\;\right]}^{-1}=e\;S_{o}\;\left[A\right]\;g_{ct\;}\left(E_{F}\right)\;k_{ct\;eff\;}\left(L\right),$$

where we introduced the effective rate constant

*k*_*eff ct*_ = *k*_*ct*_ (1+ *βL*)

reducing seemingly the number of unknown quantities for determination of the electron structure DOS function g*_ct_*(*E*). If we suppose the structure and morphology of the surface and bulk of OS are more or less identical, we may suppose for a while the identical electron structure DOS functions *g_sc_*(*E*) = *g_ct_*(*E*). Then, from Equations (3) and (4) the effective rate coefficient is

(8) $$k_{\;eff\;ct\;}\left(L\right)=\frac{L}{R_{ct}C_{sc\;}\left[A\right]}.\;$$

The effective rate constant k_*effct*_ is accessible from the measured *R_ct_ C_sc_* or fixed quantities *L* and [*A*] via Equation (8). For *βL* >> 1 it scales linearly with the sample thickness *k_eff_*_*ct* ≈_ *L*^1^ (as *R_ct_ C_sc_* ≈ const., due to the measuring principle, explanation see in [6]), for *βL* << 1 it is *k_eff ct_* = *k_ct._*

The effective rate constant *k_eff_*_*ct*_ may serve only for recalculating the measured differential CT resistance *R_ct_*(*E*_F_) to the DOS function *g*(*E*_F_), but not for finding any details about the pores’ density coefficient *β* or transfer rate *k_ct_*. 

In case we know the pores structure of the OS, characterized by the pores’ density coefficient *β*, the transfer rate constant *k*_et_ is

(9) $$k_{ct\;}=\frac{1}{R_{ct}C_{sc\;}\left[A\right]\beta }.\;$$

and the corresponding DOS function is

(10) $$g_{ct\;}\left(E_{F}\right)=e\;S_{o}\;\left[A\right]k_{ct\;}\left(1+\beta L\right)$$

The pores’ density coefficient *β* expressing the density of pores in OS, may be related to the generally recognized Apparent Brunauer-Emmet-Teller surface areas and SABET. The quantity SA, expressing the effective area of the microporous polymer per 1 g of mass [20] may be expressed as SA = *β*/ρ, where ρ is the mass density of the polymer in question. Also, the transfer rate of poreless material *k_ct_* may be a useful output of the method ER-EIS. Both unknown quantities are accessible from the thickness measurements of the impedances’ components *R_ct_*(*E*) and *C_sc_*(*E*), from Equations (7) and (8).

### 2.4. How It Is with Compensating Ions with ER-EIS?

The above-described transport of charge carriers in OS and thus its applications are in general impaired by the necessary presence of intercalation of compensating counterions. When OS layers are cycled with inappropriate electrolytes, the reversible redox switching of the film is accompanied by diffusion of counterions into the polymer matrix to maintain charge neutrality. This showed a large effect on the changes in structure, electro-activity, and long-term charge stability of applications. In their seminal papers, cited nowadays, C.E. Chidsey and R.W.Murray [31,32] derived the theory of the influence of the ionic processes on the capacitance and electronic currents in OS and gave criteria for the application of OS without ionic processes influence. In [33] the authors studied polypyrrole films by voltammetry and their results showed the conditions for the saturation of counterions concentration in the OS matrix, reached by the critical minimum scan slope of 60 mV/s. Very important in this respect were also several papers of J. Bisquert and G. Garcia-Belmonte, [34,35,36] who found, using real vs. imaginary capacitance by EIS measurements, the framework for distinguishing the minimum critical frequencies *ω*_d_, above which the ionic transport may be neglected and found also a general expression for it, *ω*_d_ $=\frac{D}{l^{2}}$, where *D* is the diffusion coefficient and *l* is the diffusion length. They found for the polypyrrole film and the thickness of 150 nm the minimum critical frequency was about *f*_d_ ≈ 10 mHz. [37,38] or by the chemistry of building in the compensating charge into backbones of polymers [39]. There is another ill effect connected with ions intercalation. The position of Fermi energy in OS is given by the interplay of both electronic and ionic shares, influencing both the Fermi energy position and even the value of the electron transfer rate function *k_ct_*(*E*) [40] The theory of the share of electronic to ionic components of transport in systems with OS was presented in [32,41]. In [42] they measured and evaluated the EIS spectra of P3HT layers after loading with the overvoltage—1.05 V (P3HT HOMO maximum) in the time interval of 0–80 min. The manifestation of the intercalation of counterions on redox properties shows a decrease in redox intensity, an increase in the CT resistance, and a decrease in the low-frequency capacitance. No changes were observed in the first 10 min of loading.

### 2.5. Materials and Apparatus

The archetypal poly(3-hexylthiophene-2,5-diyl) (P3HT) is remaining nowadays the most used conjugated polymer donor for large-scale production of organic solar [43]. P3HT is a wide bandgap donor homopolymer (1.9 eV) that consists of a repeating alkylated thiophene monomer unit. The planar nature of thiophene—thiophene linkages renders P3HT a relatively crystalline polymer with extended π-electron delocalization along its backbone. As a simple homopolymer, only one monomer is needed, the synthesis is facile and comparatively cheap to most alternative donor polymers, and P3HT has been synthesized in flow with excellent control over the molecular weight. The microstructure in P3HT and resulting electronic transport are critically affected by regioregularity [44], molecular weight [45], and samples’ preparing methods [46,47] due to the strong inclination of the homopolymer to crystallization both in a-direction (edge-on) and b-direction (face-on) stacking in film [48,49,50]. P3HT is also known to crystallize into ribbon-like nanofibers in mixed solvents or poor solvents [51] The relatively good charge transport properties, photon harvesting, and ready availability of P3HT, coupled with the excellent electron accepting properties and processability of PC61BM have led to P3HT:PC61BM becoming the most thoroughly studied system in OSCs, with over 1000 publications discussing this blend [52]. Despite the extensive research, the external power efficiency reached a maximum of 7% and the extensive research ended, for the time being, with a detailed summarizing paper [53].

The regioregular P3HT films with the thicknesses *L*ε(10; 360) nm, surface area *S* = 0.19 cm^2^ prepared by spin coating in the glovebox from the solution 1.5 wt.% P3HT in dichlorobenzene (DCB) at highly dopped c-Si substrates both p^+^ (for measurements of HOMO) and n^+^ (for measurements of LUMO) and provided by subsequent temperature and solvent annealing. The measurements electrolyte was acetonitrile (0.1 M) with the salt TBAPF6 (tetrabutylammonium hexafluorophosphate, C_16_H_36_F_6_NP) with active concentration of ions [*A*] = 610^19^ cm^−3^. 

Analytical, model 1260 (Ametek Scientific Instruments, Oak Ridge, TN, USA) was used for the ER-EIS experiment. using a three-electrode electrochemical cell with a volume of about 200 μL. The potential of the working electrode with respect to the reference Ag/AgCl electrode was controlled by the potentiostat. Pt wire was used as the counter electrode. The frequency was set to 0.5 Hz, the amplitude of the AC voltage, was 100 mV, and the sweep rate of the DC voltage ramp was 10 mVs^−1^. The measurements were performed in a glove box with a protective N_2_ atmosphere with O_2,_ and H_2_O below 20 ppm and 2 ppm, respectively. The detailed technical information is to be found in [54]) and on the ER-EIS method itself in [6].

## 3. Results

### 3.1. Pores Structure

**Figure 2:**
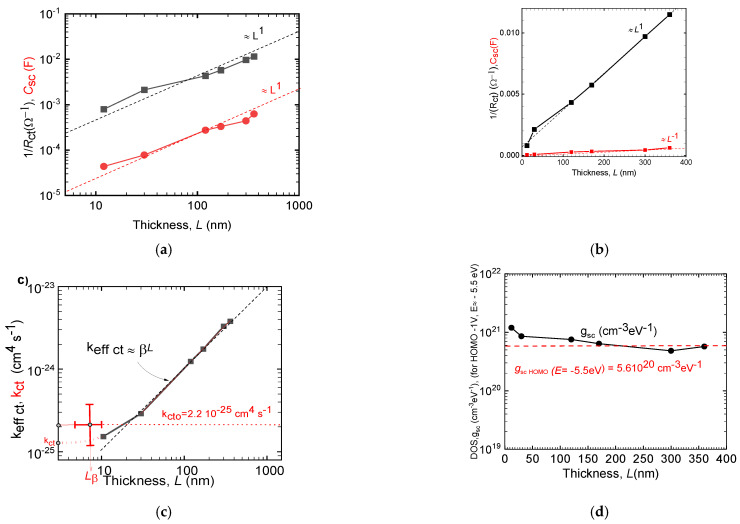
ER-EIS data of the P3HT films *L*ε(10;360)nm; (**a**) log vs. log (**b**) lin vs. lin, The rough unprocessed ER-EIS 0.1/*R_ct_* and *C_sc_* data for HOMO top (*U* =−1 V, *E* = −5.5 eV) vs. the thickness *L*; (**b**) the fitting the linear part gave *β* = 0.038 ± 0.002 nm^−1^ (and *L*_β_= 26 ± 2 nm) and k*_ct_* = 4.3 × 10^−25^ cm^4^ s^−1^; (**c**) The effective rate constant k_eff *ct*_ = k*_ct_* (1 + *βL*) (black) of the overall D-A rate vs. sample thickness *L*, also denoted is the extrapolation fitting for the poreless P3HT rate constant k*_ct_* = 1.3 × 10^−25^ cm^4^ s^−1^ (red dotted curve) and slope fitting k*_ct_*_o_ = (2.2 ± 0.6)10^−25^ cm^4^·s^−1^; (**d**) The DOS function gained using Equation (3) from the ER-EIS data (Figure S2b), g*_sc_*(for *U* = −1 V, *E* = −5.5 eV) vs. film thickness *L* with the average value *g_sc_* = 5.6 × 10^20^ cm^−3^ eV^−1^.

In Figure 2a, bare thickness dependences *L*ε(12; 360) nm of differential space-charge capacitance *C_sc_*(*L*) and differential resistance *R_ct_*(*L*) for regioregular P3HT optimized films for the maximum of the HOMO DOS, corresponding to the externally applied voltage *U* = −1 V, and the HOMO energy *E*= −5.5 eV(corresponding to the top of the HOMO band of P3HT data, Supporting Information Figure S1a). As expected, space-charge capacitance *C_sc_*(*L*) scales with the thickness *C_sc_* = f(*L*^1^) (see Equation (3)). The quantity 1/*R_ct_* also scales with thickness *L,* in contradiction to the expectations of Marcus’ interface recombination theory (Equation (4)) [15]. The sample thickness *L* may be introduced into the original Marcus’ theory via the existence of the pores by increasing surface area *S* = *S*_o_(1 + *Lβ*) (Equation (6)). In principle, the linear dependence 1/*R_ct_* = f(*L*^1^) where the slope is increasing with the coefficient *β* and intersection increasing with the transfer rate *k_ct_* may give both quantities with limited precision, in this case, volume density of pores was determined as *β* = 0.038 ± 0.002 nm^−1^ (and *L*_β_ = 26 ± 2 nm) and *k_ct_* = 4.3 × 10^−25^ cm^4^ s^−1^.

In Figure 2c is the dependence of the effective transfer rate coefficient *k_eff_*_*ct*_, (from Equation (7) on the sample thickness *L*, in the used interval of thicknesses *L*ε(10; 360) nm, being far from a constant, rising about 30 times with thickness *L*. The effective transfer rate coefficient *k_eff_*_*ct*_ = *k_ct_* (1 + *βL*) for *βL* < 1 should approach *k_eff ct_* = *k_ct_* (dotted red curve) *k_ct_* ≈ 1.3 × 10^−25^ cm^4^ s^−1^ and slope fitting *k_cto_* = 2.2 × 10^−25^ cm^4^ s^−1^. In Figure 2d are calculated the DOS values for *E* = 5.5 eV, for all the samples of variable thickness *L* from ER-EIS data of the measured differential capacitance *C_sc_*, using Equation (3). All films gave identical values with a reasonable deviation from the average value g = 5.6 × 10^20^ cm^−3^ eV^−1^, testifying the electron structure and the DOS in P3HT films are not dependent on the sample thickness in the range *L*ε(12; 360) nm (see Figure 2d and Figure S2 in the Supporting information). This Experimental result is of utmost importance for the branch, as it gives the method for clarifying the apparent thickness dependence of various quantities. Of excessive importance for the ER-EIS method is the result for proving the basic condition for ER-EIS existence, i.e., small enough voltage on the polymer compared with the applied voltage. The spectra are not shifted for samples with thicknesses quotient exceeding 1:30, with small enough currents, but differing by orders of magnitude. The measurements give extremely low SA= *β*/ρ = (10^3^ 0.14/1.3) = 34.5 m^2^g^−1^, where ρ = 1.1 g cm^−3^ is the mass density of rr P3HT [55].

### 3.2. Rate Equations for the Bimolecular Preexponential Rate of Heterogeneous Interface Electrolyte/OS

Let us deal with a rather complicated heterogeneous interface electrolyte/OS, having in mind the R.A. Marcus D-A CT theory for electron transfer with the little spatial overlap of the electronic orbitals of the two reacting molecules described by the framework of the electrode reaction of elementary steps in series with the total reaction rate determined by a single, slowest step. The author wants to stress, that the following notes only touch the rather demanding problem, without any ambitions to solve it, limiting the note only to the guess of the order of the preexponential term of the total CT D-A rate and highlighting the reasons for it. 

**Figure 3:**
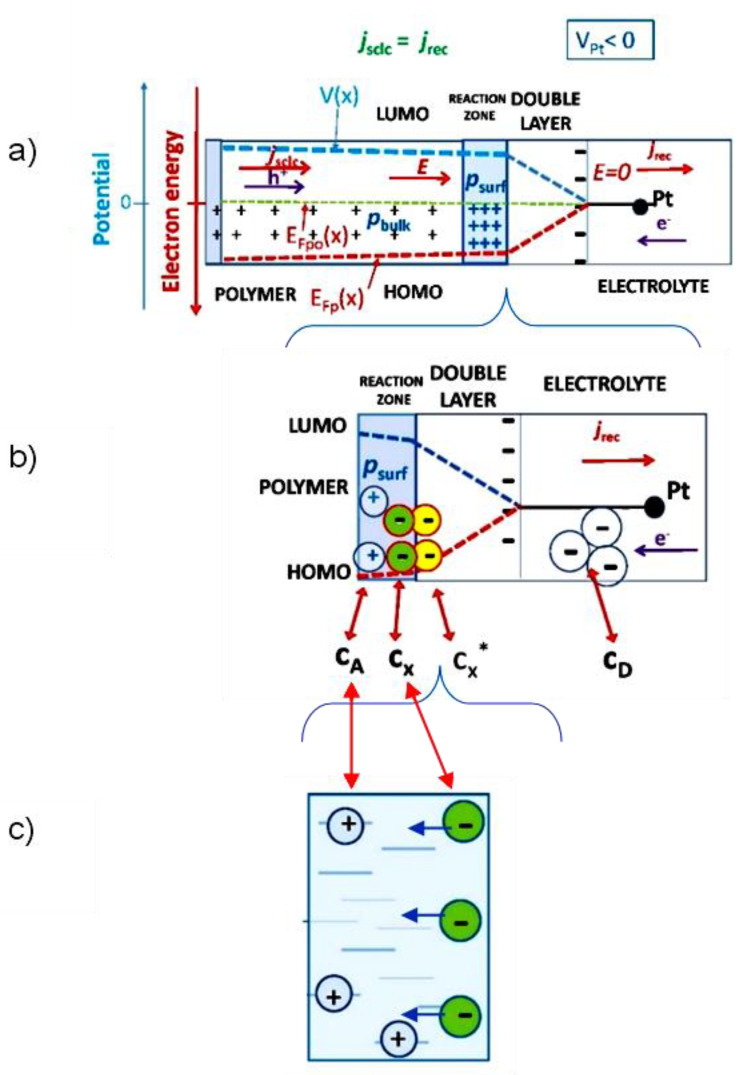
(**a**) Steady-state charge transport process in electrolyte/OS system for *V*_Pt_ < 0 in OS with the established Fermi energy *E*_Fp_, the positively charged OS surface layer (reaction zone) with the surface concentration of holes *p*_surf_. To support the current via the system, the HOMO surface concentration of holes *p*_surf_ is neutralized (reduced) by electronic s incoming from the electrolyte, forming the recombination current *j*_rec_. The created electric field strength *E* in the OS bulk is followed by the injected compensating holes from back contact, forming bulk current in the OS *j*_SCLC_; (**b**) Enlarged “reaction” zone on the surface of OS where electronic s travel by hopping, discharging localized holes, which is enlarged in panel (**c**).

Let us resort to the first years of electrochemical CT theory of R.A. Marcus [56]. The electrochemical system depicted in Figure 3a, and depicted in increasing detail in Figure 3b,c, may be described by rate equations of the reaction scheme, describing the overall bimolecular reaction *A* + *D* ↔ products

(11) $$A+D{↔}_{\;k_{-1}}^{\;k_{1}}\;X^{*}$$

(12) $$X^{*}{↔}_{\;k_{-2}}^{\;k_{2}}X\;$$

(13) $$X{↔}^{k_{3}}\;\mathrm{products}$$

For pure diffusion governed CT in steady-state R. A. Marcus realized that for a slight overlap of the electronic orbitals of the two reacting particles (donor D and acceptor A, see Figure 3b before an electron can jump from one reactant to the other, there has to be first a “reorganization”, expressed by the reorganization energy, of the molecules around both ions and such that the total energy of the entire system before and after the electronic jump was unchanged and energy was conserved in the jump. Accordingly, it was possible to formulate the rate equations with corresponding concentrations of donors c_D_, acceptors c_A_, and final products. The reaction proceeded by way of two successive intermediate states X* and *X* with concentrations *c_x_* and $c_{x}^{*}$, which have the same atomic configurations but for different electronic configurations and equal energy. In our case X* state is represented by an electron at the surface of the interface, and *X* by the electron in the region of the reaction zone of the OS.

The overall rate constant *k_ct_* of this reaction sequence fulfills

(14) $$k_{ct\;}c_{A}c_{D}=k_{3\;}c_{x}.$$

The steady-state equations for the individual steps fulfill

(15) $$\frac{dc_{x}^{*}}{dt}=0=k_{1\;}c_{A}c_{D}-\left(k_{-1\;}+k_{2}\right)c_{x}^{*}+k_{-2}c_{x},$$

And

(16) $$\frac{dc_{x}}{dt}=0=k_{2\;}c_{x}^{*}-\left(k_{-2\;}+k_{3}\right)c_{x},$$

Equations (14)–(16) give for the steady-state value of the overall rate constant

(17) $$k_{ct\;}=\frac{k_{1\;}\;}{\left[1+\left(1+\frac{k_{-2}}{k_{3}}\right)\frac{k_{-1}}{k_{2}}\right]\;}.$$

The overall bimolecular rate constant *k_ct_* for the **diffusion governed CT process** in the system electrolyte/crystalline semiconductor, where individual rate constants fulfill $k_{3}>>k_{-1}$ and also $k_{2}>k_{-1\;}$ is [56]

(18) $$k_{ct\;}≂k_{1},$$

measured for several crystalline semiconductors in the range *k_ct_* ε (10^−16^;10^−18^) cm^4^ s^−1^ [19].

The overall rate of the bimolecular transfer process D-A is decisively dependent on the rate constant *k*_3,_ which is the slowest process in the whole sequence of redox processes [57]. If it is valid for individual rate constants $k_{3}<<k_{-1},\;k_{2},k_{-2}$ the **process is reaction limited** with the overall all bimolecular rate constant

(19) $$k_{ct}=k_{1}\frac{k_{3}}{k_{-1}\;}.\;$$

For the interface electrolyte/OS the active donors with concentration *c_D_*, are dispersed in the electrolyte and travel by the diffusion process towards the interface and reaction zone, which is occupied by the positively charged holes with concentration *c*_A_, residing at the localized states of HOMO. Figure 3b,c is depicted the hopping neutralization (reduction) process of excess holes, acting as acceptors. The whole discharging process is limited by the CT rate of hopping k_3_ [2] in the “reaction zone”, estimated in several works to be about 1 nm [56]. In [58] based on the full quantum treatment of charge dynamics in amorphous molecular semiconductors with the inclusion of full quantum phonon modes, they came to k_3_ rates in typical disordered OS, spanning in the interval *k*_3_ϵ (10^6^–10^10^) s^−1^, with the important note on the necessity of visiting a state with the longest time once, forming decisively the overall rate. Taking *k*_−1_ = 10^13^ s^−1^, *k*_2_ = 2.10^12^ s^−1^, *k*_−2_ = 2.10^12^ s^−1^ [56] and *k*_3_ = 10^6^ s^−1^ [58] we obtain for the overall bimolecular transfer rate for the transport limited D-A CT process in OS.

(20) $$k_{ct}=\frac{k_{1\;}k_{3\;}}{k_{-1\;}}=\frac{10^{-18\;}10^{6}}{10^{13}}≅\;10^{-25}\;{\mathrm{cm}}^{4}\;s^{-1},$$

where we used *k*_1_ = 10^−18^ cm^4^ s^−1^ the value for the overall diffusion process in the crystalline semiconductor [59]. 

### 3.3. Counterions Intercalation 

One of the goals of the present paper was also to elucidate the conditions for the correctness of the DOS spectroscopic method ER-EIS [11,60] concerning the intercalation of counterions. The voltage scan slopes used in the ER-EIS method are in the range of 10 mV/s, corresponding to about ≈10 mHz and the total time for one run is comparatively short, for voltages Uε(0–1(2)) V it is tε(100–200) s. Thus, the errors on the energy axis by neglecting the counterions influence and on the measured signals, caused predominantly by the shift of the Fermi energy and morphological changes, maybe both neglected. 

On top of this, as pointed out by Arkhipov [61], a high carrier density can be reached without introducing counter charges due to a high level of monopolar charge injection via a contact (forming SCLC), resulting in charging of the volume of OS by charge carriers of one sign. Under these circumstances, the Coulomb interaction between carriers of the same sign cannot create coulomb traps and thus is much weaker, resulting in a much smaller broadening of the DOS distribution.

## 4. Discussion and Conclusions

The present paper deals with three often mentioned seeming obstacles in experimental praxis with ER-EIS with OS, namely the ill influence of the omnipotent pores structure, the ill influence of the penetration into the structure of the charge compensating ions, and a very important question of the involvement of the Marcus’ CT D-A transport theory in the ER- EIS method. Let us summarize. 

Omnipotent and universal pores’ existence in polymers is shared knowledge. First of all, it is important to state, that the pores and pore structure need not influence the spectroscopic properties of the ER-EIS method, if properly evaluated using the measured capacitance *C_sc_*, while the space-charge capacitance *C_sc_* is in no way influenced by pores’ existence and evaluation of the electron structure i.e., the DOS function *g_sc_*(*E*) via the space-charge capacitance *C_sc_*(*E*) and Equation (3) is universally valid. The second measured component *R_ct_*(*E*) (Equation (4)) cannot be used for the evaluation without the knowledge of the effective transfer rate constant *k_ct_*_*eff*_(*E*).

The results of the ER-EIS show that taking into consideration the electrolyte penetration process is rather fast, completed fully for *L* = 120 nm thick sample in 10 s [62] so for all examined thicknesses was fully accomplished. Then, the results of calculated ER-EIS DOS function *g_ct_*(*E*) for all samples merge almost into one plot, achieving for the examined point of the HOMO top the value g_HOMO_ (*E* = −5.5 eV) = 5.610^20^ cm^−3^ eV^−1^, independent of the sample thickness (Figure 2d and Supplement Information Figure S2a)). From this follows the next important result—the voltage at the OS bulk of the film irrespective of its thickness *L* was negligible compared to the external voltage *U* and thus the energy axis for all the measurements were correct. This constitutes important proof for the ER-EIS method outputs. 

The ER-EIS thickness measurements provide two new quantities, the pores’ density coefficient of the pores *β*(nm^−1^) and the overall transfer rate Marcus’ constant of the electrolyte/OS system, *k_ct_* (cm^4^ s^−1^) of the corresponding pore less solid. For the examined OS films of P3HT both quantities are *β* = 0.038 ± 0.002 nm^−1^ (and *L*_β_ = 26 ± 2 nm) and *k_ct_* = 4.3 × 10^−25^ cm^4^ s^−1^, though loaded with considerable uncertainty due to a small number of data (Figure 2b) and its cumulative value gained from all data *k_ct_* = (2.2 ± 0.6) × 10^−25^ cm^4^ s^−1^ (Figure 2c). 

For the attempt to the explanation of the electronic D-A CT transport via the interface electrolyte/OS and to obtain the bimolecular rate constant, we used the seminal paper of R.A. Marcus [56,63] and the contemporary knowledge of the charge transport in OS, where the mechanism of compensating charges from negative donors are brought to the interface with OS, transferred to the electronic form, and discharging by hopping transport the positively charged localized states, situated at the depth of about 1 nm from the interface [19,64]. 

The second decisive circumstance in the final result is a wide distribution of hopping times in OS with a necessary extremely long hopping rate constant, reaching 10^6^ s^−1^ [2], leading to the prevailing of the reaction dominated transport in OS with a low reaction rate compared to the much faster diffusion dominating rate constants. This leads to the low resulting value of the bimolecular rate constant *k_ct_* = 10^−25^ cm^4^ s^−1^ in approximate, but reasonable accordance with the results achieved by the ER-EIS method.

Intercalation of compensating ions for the present set-up of the method ER- EIS is not endangering the results due to the effective frequency of the ramp used and the perturbation of AC signal frequency. 

In conclusion:

1. The ER-EIS method may be used with advantage in the field of OS for the elucidation of:
  - The electronic structure (DOS) and statistics of occupation of transport bands (HOMO and LUMO),
  - The electronic structure of both intrinsic and extrinsic defect states,
  - The electronic structure of excited states in D-A systems, so crucial for organic solar-cell functioning,
2. The effect of injection of high current densities by redox interface reactions in the bulk of OS with built-in pores structure may be very interesting for the design of new devices of organic electronics and energy storing.
3. The present paper dispelled concerns associated with the use of the method, due to the pore structure existence and penetration of ions. It may be also concluded the ER-EIS method is not in any contradiction with Marcus’ CT D-A transport theory.

## Data Availability

Data are available from the author, fully comply with the data Availability Statements in section “MDPI Research Data Policies” and stem from the long-lasting and successful cooperation and co-authorship of the author from the Faculty of Informatics, Tomas Bata University in Zlin, Czech Republic, and the Institute of Physics, Slovak Academy of Sciences in Bratislava Slovakia.

## Supplementary Materials

The following supporting information can be downloaded at: https://www.mdpi.com/article/10.3390/polym14173456/s1, Figure S1: The data of the ER-EIS on P3HT both differential capacitance Csc(E) (a) and differential resistance Rct(E) (b); Figure S2: The electron structure DOS function *g_sc_*(*E*) of P3HT films.
