# Supplementary material for: Measuring the Pores’ Structure in P3HT Organic Polymeric Semiconductor Films Using Interface Electrolyte/Organic Semiconductor Redox Injection Reactions and Bulk Space-Charge
Source: Polymers (Basel). 2022 Aug 24;14(17):3456. doi: 10.3390/polym14173456 (PMC9460914; doi:10.3390/polym14173456)
Supplement: Supplementary file 1 [file polymers-14-03456-s001.zip › polymers-1825136-supplementary.pdf]

Supplementary Materials

Measuring the Pores' Structure in P3HT Organic Polymeric Semiconductor Films Using Interface Electrolyte/Organic Semiconductor Redox Injection Reactions and Bulk Space-Charge

Franz Schauer

Faculty of Applied Informatics, Tomas Bata University in Zlin, Nad Stranemi 4511, 760 05 Zlin, Czech Republic; fschauer@fai.utb.cz

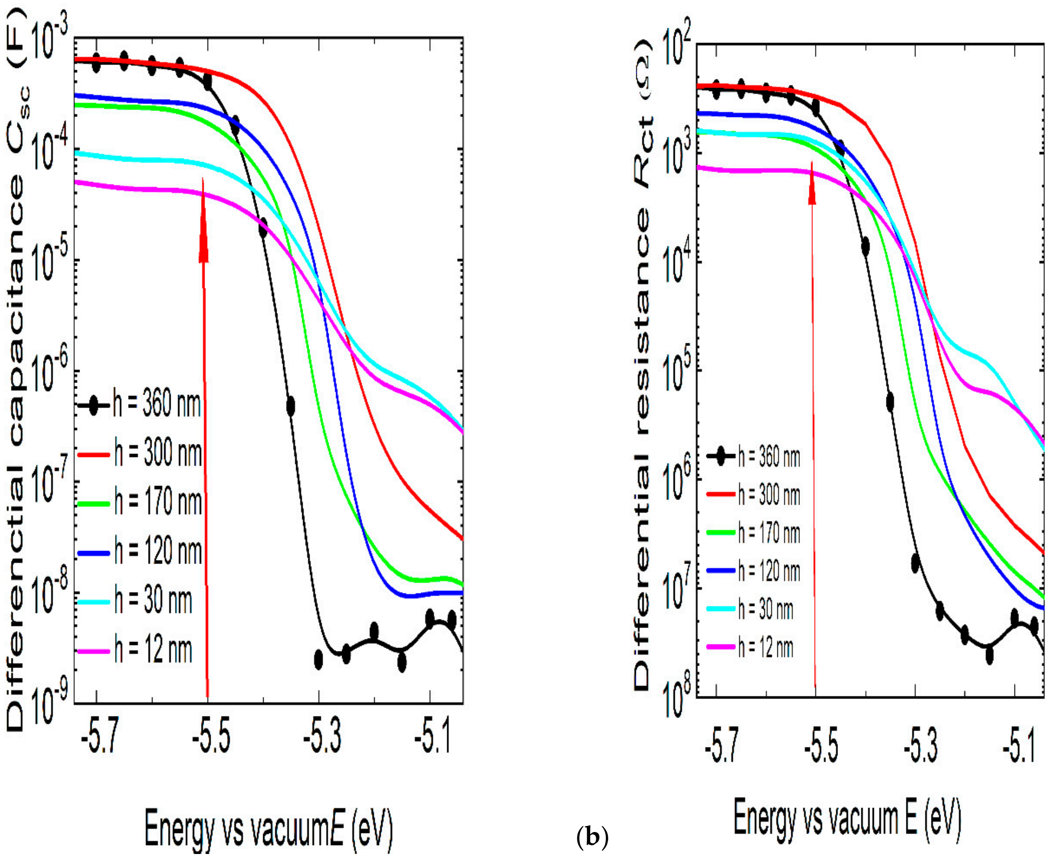

(**a**) (**b**)

Figure S1. The detail of the measured data of P3HT films with thickness L ε (12; 360) nm, the differential capacitance $C_{sc}(E)$ (**a**) and the differential resistance R_{ct} (E) (**b**).

Rough, unprocessed data of the ER-EIS method for P3HT rreg film; the parameter is the film thickness, L ε(12; 360) nm; (a)The differential resistance R_{ct} (E) ; (b) The differential capacitance $C_{sc}(E)$; The arrow depicts the energy of the maximum of the HOMO band (5.5 eV), where the data were taken for the detailed evaluation for the pores structure (see Figure 2).

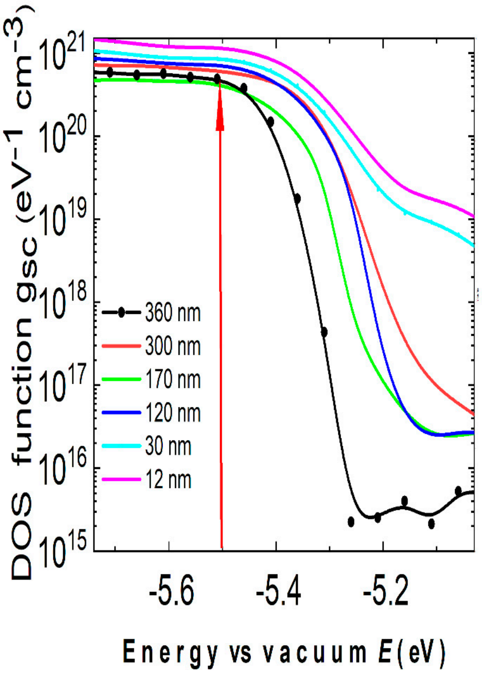

(**a**)

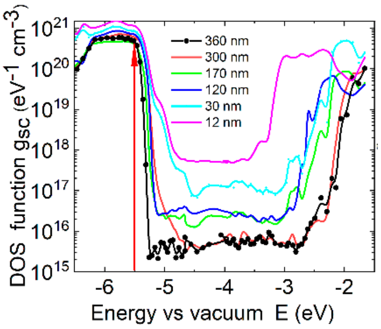

(**b**)

Figure S2. The electron structure DOS function g_{sc} (E) (from the $C_{sc}(E)$ data in Figure S1a.

The electron structure DOS function of the P3HT films g_{sc} (E); (a) Detail of the HOMO; the arrow depicts the energy of the maximum of the HOMO band (5.5 eV), where the data were taken for the detailed evaluation of the pores structure in the article (see Figure 2); (b) The total energy range HOMO-LUMO of the electron structure function g_{sc} (E) of the examined P3HT films.
